# Two cases of neonatal hyperglycemia caused by a homozygous *COQ9* stop‐gain variant

**DOI:** 10.1111/jdi.70022

**Published:** 2025-03-10

**Authors:** Russell Donis, Maryam Al Badi, Nadia Alhashmi, Andrew T Hattersley, Sarah E Flanagan, Elisa De Franco

**Affiliations:** ^1^ Department of Clinical and Biomedical Sciences, Faculty of Health and Life Sciences University of Exeter Exeter UK; ^2^ National Diabetes and Endocrine Centre Muscat Oman; ^3^ National Genetic Centre, Royal Hospital Muscat Oman

**Keywords:** Coenzyme Q10, Mitochondrial disease, Neonatal diabetes

## Abstract

Neonatal diabetes mellitus (NDM) is a monogenic condition diagnosed <6 months of age with >40 genetic causes. International guidelines recommend referral for genetic testing immediately after diagnosis since the genetic result guides clinical management. We used next‐generation sequencing to identify a homozygous pathogenic variant, p.(Arg244*), in *COQ9* in 2 individuals referred for NDM testing. Both had insulin‐treated hyperglycemia, severe structural brain defects, dysmorphic features, and lactic acidosis. Recessive loss‐of‐function variants in *COQ9* cause Coenzyme Q10 deficiency‐5, a multi‐system mitochondrial disease, with 7 cases reported. Neonatal hyperglycemia has not been reported in any of these cases but has been described for two other Coenzyme Q10 disorders caused by variants in *COQ2* and *COQ4*. Our report shows that individuals with *COQ9*‐related disease can present with neonatal hyperglycemia, expanding the clinical spectrum of this disorder. We recommend the inclusion of *COQ9*, as well as *COQ2* and *COQ4*, to gene panels used for NDM testing.

## Abstract 2

NDM is a monogenic condition diagnosed <6 months. We present the first two cases of individuals with pathogenic variants in COQ9 who presented with neonatal hyperglycemia. COQ9 should be included on NDM genetic testing panels.

## INTRODUCTION

Neonatal diabetes mellitus (NDM) is a monogenic condition diagnosed in the first 6 months of life. Over 40 different genetic aetiologies have been described in individuals with NDM^1^, explaining >85% of cases^2^.

A genetic diagnosis guides clinical management in individuals with NDM and determines which treatment is most appropriate (for example, sulphonylureas in individuals with activating variants in the potassium channel genes *KCNJ11* ^3^, ^4^ and *ABCC8* ^5^, ^6^), and whether the diabetes occurs in isolation (isolated NDM) or is one of the first manifestations of a multiorgan disease (syndromic NDM). The genes involved in the pathogenesis of syndromic NDM have essential roles in biological pathways; for example, *GATA6*, crucial in organ development^7^, and *EIF2AK3*, crucial for the endoplasmic reticulum stress response^8^.

Recent findings have highlighted mitochondrial dysfunction as another mechanism leading to NDM with the identification of novel NDM aetiological genes, such as *NARS2* ^9^ and *TARS2* ^10^, both encoding proteins essential for mitochondrial protein translation. The importance of mitochondrial function to beta cells is further supported by previous studies identifying recessive variants in *CYC1*, which encodes a subunit of Complex III of the mitochondrial respiratory chain^11^, ^12^ and mitochondrial DNA deletions causing Pearson syndrome in 3 and 6 individuals with NDM, respectively^13^. Furthermore, the m.3243A>G variant of the mitochondrial DNA is a cause of later onset diabetes in individuals affected by Maternally inherited diabetes and deafness (MIDD)^14^.

International consensus guidelines recommend that all infants diagnosed with diabetes in the first 6 months of life be immediately referred for genetic testing^15^. Targeted sequencing analysis of a panel including all the known NDM genes^16^ is the most common approach.

Here, we describe two individuals who were referred for NDM genetic testing following presentation with neonatal hyperglycemia and extra‐pancreatic features. In both individuals, a homozygous stop‐gain variant was detected in *COQ9*, a gene essential for mitochondrial function, which is not routinely included on NDM gene testing panels.

## MATERIALS AND METHODS

Probands 1 and 2 had hyperglycaemia detected on the first and third days of life, respectively, which required insulin treatment. Additional clinical details are summarized in Table 1 and in the Appendix S1.

**Table 1:** Clinical features of individuals reported with pathogenic *COQ9* variants

	Proband 1 (this study)	Proband 2 (this study)	Proband 3^19^	Proband 4^27^	Proband 5.1^28^	Proband 5.2^28^	Proband 5.3^28^	Proband 5.4^28^	Proband 6^29^
*COQ9* variant	p.(Arg244*) c.730C>T homozygous	p.(Arg244*) c.730C>T homozygous	p.(Arg244*) c.730C>T homozygous	p.(Ser127_Arg202del) c.521 + 1del homozygous	p.(Ser127_Arg202del) c.521+2T>C p.(Ala203_Asp237del) c.711+3G>C compound heterozygous	p.(Ser127_Arg202del) c.521+2T>C p.(Ala203_Asp237del) c.711+3G>C compound heterozygous	p.(Ser127_Arg202del) c.521+2T>C p.(Ala203_Asp237del) c.711+3G>C compound heterozygous	p.(Ser127_Arg202del) c.521+2T>C p.(Ala203_Asp237del) c.711+3G>C compound heterozygous	p.(Gly129Valfs) c. 384delG homozygous
Age at clinical presentation	1 day	1 day	6 h	Birth	19 weeks gestation	23 weeks gestation	22 weeks gestation	19 weeks gestation	Birth
Age and cause of death	Before 3 weeks, respiratory failure	6 weeks, seizures and cyanosis	2 years, chest infection	18 days, cardio‐respiratory failure	3 days	Pregnancy termination at 28 weeks	12 h at extubation	Pregnancy termination at 19 weeks	Alive, 3 years
IUGR (bwt, gestation)	Yes (1,940 g 37 weeks)	Yes (1,660 g 39 weeks)	Unknown	Yes (1,440 g 36 weeks)	Yes (1,460 g 33 weeks)	Yes (bwt unknown, 28 weeks)	Yes (1,580 g 34 weeks)	Yes (bwt unknown, 19 weeks)	Yes (2000 g 38 weeks)
Oligohydramnios	Not reported	Yes	Not reported	Yes	Yes	Yes	Yes	Not reported	Yes
Hyperglycemia	Detected day 1	Detected day 3	Not reported	Not reported	Not reported	n/a	Not reported	n/a	Not reported
Metabolic features	Not reported	Elevated lactate	Lactic acidosis	Increased alanine, lactic acidosis	Elevated lactate	n/a	Elevated lactate	n/a	Increased alanine, elevated lactate
Microcephaly	Not reported	Yes	Yes	Yes	Yes	n/a	Yes	n/a	Yes
Arthrogryposis	Yes	Yes	Not reported	Yes	Yes	n/a	Not reported	n/a	Yes
Brain imaging findings	Cerebellar and optic hypoplasia	Dandy‐Walker malformation	Cerebral and cerebellar atrophy	Leigh‐like syndrome	Hydrocephalus, lobulated cysts in frontal lobes	n/a	n/a	n/a	Cerebellar hypoplasia vermis and brain stem, enlarged lateral ventricles, corpus callosum agenesis, cortical atrophy
Neuro‐developmental features	Seizures	Seizures	Seizures, developmental delay, dystonia	Seizures, hypotonia	Not reported	Not reported	Abnormal jerking movements, autopsy showed signs of Leigh syndrome	Not reported	Seizures, developmental delay, hypotonia
Additional features	Hypertrophic cardiomyopathy	Umbilical hernia, dysmorphic features, increased cortical echogenicity in kidneys, pulmonary hypertension, small ventricular septal defect	Poor feeding, hypothermia, increased tone, renal tubulopathy, left ventricular hypertrophy	Poor respiratory efforts, generalized cyanosis, recurrent apnea and bradycardia	Pulmonary hypertension, respiratory depression, fixed dilated pupils, brisk reflexes, dilated ventricles heart, bradycardia, cardiomegaly, dysmorphic features	Echogenic bladder, large cystic kidneys	Hypotension, echogenic bowel, dysmorphic features	Echogenic bowel and kidneys	Respiratory distress, cardiomyopathy, cystic kidneys, dysmorphic features

bwt, birthweight; n/a, not applicable.

DNA samples from both probands and the parents of proband 1 were sent to the Exeter genomics laboratory for NDM genetic testing. Samples from proband 2's parents and deceased sibling were not available for testing. All known genetic causes of NDM were tested in both probands using a combination of Sanger sequencing, targeted next generation sequencing (tNGS^16^) and methylation‐specific PCR to assess the imprinted chr6q24 transient neonatal diabetes region. Nine years after the original referral, whole exome sequencing (WES) was performed on the samples from proband 1 and her parents to identify potential novel genetic causes of NDM. The study complied with the Declaration of Helsinki, with informed consent obtained from the parents.

Exome sequencing and targeted next‐generation sequencing were performed as previously described^7^, ^16^. An in‐house script was used to prioritize rare (allele frequency in GnomADv4^17^ <0.001%) *de novo*, homozygous, and compound heterozygous coding variants. *De novo* variants were considered only if the ratio between the reads supporting each allele was >0.2. Variant classification was carried out using the ACMG guidelines^18^.

After the identification of a likely genetic cause in Proband 1, replication studies were undertaken to search for additional cases. RNA baits targeting the 9 coding exons and intron/exon boundaries of the *COQ9* gene (NM_020312.3) were added to our custom‐designed tNGS panel^16^. Details of the RNA baits are available upon request. The replication cohort consisted of 168 individuals with genetically unsolved NDM, which included proband 2. Due to privacy concerns, the research data supporting this publication are not publicly available.

## RESULTS

Exome sequencing data in Proband 1 identified only 1 variant which matched all the filtering criteria: the homozygous c.730C>T, p.(Arg244*) stop‐gain variant in *COQ9*. Both unaffected parents were heterozygous for the variant. Replication studies performed on 168 individuals with genetically unsolved NDM identified the same homozygous p.(Arg244*) variant in the sample from proband 2. Both probands were reported to be of Pakistani origin but were not known to be related. No further disease‐causing variants in *COQ9* were identified in the remaining 167 individuals.

The *COQ9* gene encodes Coenzyme Q9, a mitochondrial protein involved in the biosynthesis of Coenzyme Q10 (CoQ10), a critical component of the electron transport chain in the synthesis of ATP. Biallelic loss‐of‐function variants in *COQ9* cause Primary Coenzyme Q10 deficiency 5 (COQ10D5; OMIM: 614654), a multiorgan condition with features including intrauterine growth retardation (IUGR), microcephaly, neurological and cardiovascular disease, and metabolic lactic acidosis.

The p.(Arg244*) variant identified in the two probands in this study was classified as pathogenic according to the ACMG guidelines. This variant results in the insertion of a premature stop codon in exon 7 of 9 of the *COQ9* gene, and it is therefore predicted to cause loss of the COQ9 protein through nonsense‐mediated decay of the mRNA transcript. The p.(Arg244*) variant has been previously reported in 1 individual with COQ10D5^19^ and it is listed in ClinVar as ‘Pathogenic’ (Variation ID: 431, 3 submissions). Furthermore, studies performed on an open muscle biopsy and cultured fibroblasts from a previously reported individual with the same homozygous p.(Arg244*) *COQ9* variant showed a strong quantitative deficit of CoQ10 and significantly reduced CoQ10 biosynthesis^19^. This result confirmed a diagnosis of COQ10D5 in Probands 1 and 2.

## DISCUSSION

We report the identification of a homozygous p.(Arg244*) loss‐of‐function variant in *COQ9* in two individuals referred for NDM genetic testing, following presentation with hyperglycemia in the first 3 days of life. Both probands had severe structural brain defects, IUGR, and arthrogryposis. The clinical features observed in both individuals were consistent with the genetic diagnosis of COQ10D5.

Our results expand the phenotypic spectrum caused by recessive *COQ9* variants. Hyperglycemia has not been previously reported in any of the 7 (3 probands and 4 siblings) individuals with COQ10D5 caused by biallelic loss‐of‐function *COQ9* variants described in the literature (Table 1). Probands 1 and 2 in our study had hyperglycaemia on the first and third day of life, respectively, which required insulin treatment. In proband 2, the hyperglycaemia was transient, requiring insulin treatment until the age of 5 weeks. This individual was off insulin for 1 week before their death at 6 weeks. In proband 1, the hyperglycaemia persisted until their death at the age of 3 weeks. Since both individuals died in the neonatal period, we do not know whether the difference in insulin requirement reflects a genuine variability in the phenotype between these two patients, or whether Proband 1's insulin requirement might have also diminished with age. It is not known whether hyperglycaemia was measured in the 7 previously reported patients with *COQ9* variants, nor is it known if the one surviving individual who was 3 years of age at the time of reporting had hyperglycaemia.

Neonatal hyperglycaemia has been reported as a feature in 7 individuals with COQ10D resulting from *COQ2* (*n* = 5) or *COQ4* (*n* = 2) pathogenic variants^20^, ^21^, ^22^, ^23^. In all these cases, neonatal hyperglycemia was a presenting feature of the disorder detected before the age of 6 months (mean age of diagnosis: 63.9 days, range 1–150 days). A transgenic mouse model harboring a homozygous *Coq9* truncating variant showed significant loss of ATP and respiratory complex I activity in neuronal cells leading to encephalomyopathy^24^, which is consistent with the phenotype observed in COQ10D5 patients. No functional studies have been performed to assess the effect of pathogenic variants in *COQ2*, *COQ4*, or *COQ9* in pancreatic β‐cells.

An early genetic diagnosis of COQ10D is crucially important as CoQ_10_ replacement therapy may be beneficial in some patients^25^. The genetic cause of NDM had remained undetermined in our two patients following routine testing, as *COQ9* was not included on the tNGS gene panel, resulting in a delay in their genetic diagnosis. Many laboratories are now adopting whole exome and whole genome sequencing as first‐line genetic tests for conditions such as NDM, and rapid genome sequencing has proven particularly beneficial for individuals with mitochondrial disorders^26^. These approaches will benefit patients such as those reported in this study as they will allow comprehensive testing of a wider panel of genes such as *COQ9*.

In conclusion, we report neonatal hyperglycaemia as a presenting feature of COQ10D5 in two individuals with a homozygous loss‐of‐function variant in *COQ9*. We recommend that analysis of the CoQ_10_ genes previously associated with NDM, *COQ2, COQ4*, and now *COQ9*, is included in genetic testing panels for NDM.

## DISCLOSURE

The authors declare no conflict of interest.

Approval of the research protocol: The protocol for this research project has been approved by the Genetic Βeta‐Cell Research Bank at the University of Exeter (Ethics approval 517/WA/0327).

Informed consent: The study conforms to the provisions of the Declaration of Helsinki, with written, informed consent obtained from the parents of the subjects. Only anonymized data was used in this report.

Registry and the registration number of the study/trial: N/A.

Animal studies: N/A.

## Supporting information


**Appendix S1.** Supplementary Material.
