# Supplementary material for: Two cases of neonatal hyperglycemia caused by a homozygous COQ9 stop‐gain variant
Source: J Diabetes Investig. 2025 Mar 10;16(5):959–63. doi: 10.1111/jdi.70022 (PMC12057368; doi:10.1111/jdi.70022)
Supplement: Supplementary file 1 — Appendix S1. Supplementary Material. [file JDI-16-959-s001.docx]

**Supplementary Material**

*Clinical descriptions of 2 probands with pathogenic COQ9 variants*

Proband 1 was a female and the first child born to healthy parents who were not known to be related. She was born at 37 weeks with a birthweight of 1940g (Z-score: -2.20) following a pregnancy complicated by intrauterine growth restriction (IUGR) and oligohydramnios. She presented at birth with seizures, arthrogryposis, umbilical hernia, and cerebellar and optic hypoplasia. On the first day of life, glucose measurements detected hyperglycemia which was managed with subcutaneous insulin infusion. Hyponatraemia and hypokalaemia were also observed. An echocardiogram suggested hypertrophic cardiomyopathy. On the 8^th^ day of life, as hyperglycaemia had persisted, blood samples from the baby and both parents were collected and sent for NDM genetic testing. The child died before the age of 3 weeks due to respiratory failure.

Proband 2 was a female born at 39 weeks gestation following an uneventful pregnancy. Both parents were healthy and were not reported to be related. A previous child of the couple had died on the first day of life. The cause of their death was unknown, although a high level of lactate level had been recorded. At birth, the proband weighed 1660g (Z-score: -4.16), and microcephaly, arthrogryposis and coarse facial features were noted. On the 3^rd^ day of life, hyperglycaemia was detected (blood glucose = 25 mmol/L, C-peptide 84 pmol/l (normal range 260 – 1270 pmol/l)) and insulin treatment was started (0.5U/kg/day). On the 14^th^ day of life hyperglycaemia was persisting which prompted referral for NDM genetic testing. At the age of 5 weeks, normoglycaemia was observed and insulin treatment was discontinued. Subsequent metabolic tests revealed elevated lactate, and a mitochondrial disorder was suspected.
